# Novel Models for the Prediction of Left Atrial Appendage Thrombus in Patients with Chronic Nonvalvular Atrial Fibrillation

**DOI:** 10.1155/2019/1496535

**Published:** 2019-08-25

**Authors:** Do Van Chien, Pham Thai Giang, Pham Truong Son, Le Van Truong, Pham Nguyen Son

**Affiliations:** Department of Cardiology, Military Heart Institute, 108 Central Military Hospital, Hanoi, Vietnam

## Abstract

Predicting left atrial appendage thrombus (LAAT) in chronic nonvalvular atrial fibrillation remains challenging despite the fact that several predictive models have been proposed to date. In this study, we sought to develop new and simpler models for LAAT prediction in chronic nonvalvular atrial fibrillation. The study enrolled 144 patients with chronic nonvalvular atrial fibrillation who underwent transesophageal echocardiography for LAAT detection. We examined the association of LAAT incidence with the CHA_2_DS_2_-VASc score and echocardiographic parameters pertaining to the left atrium (LA), including diameter, volume index, strain, and strain rate measured on speckle tracking echocardiography. LAAT was found in 24.3% of patients (39/144). The following parameters had good diagnostic performance for LAAT: LA volume index >57 mL (area under the curve (AUC), 0.72; sensitivity, 77.1%; specificity, 64.2%), LA positive strain ≤6.7% in the four-chamber view (AUC, 0.84; sensitivity, 77.1%; specificity, 77.1%), and LA negative strain rate >−0.73 s^−1^ in the four-chamber view (AUC, 0.83; sensitivity, 85.7%; specificity, 70.6%). The CHA_2_DS_2_-VASc score alone had a low predictive value for LAAT in this population (*χ*^2^ = 3.53), whereas the combination of CHA_2_DS_2_-VASc score with LA volume index had significant association and better predictive value (*χ*^2^ = 12.03), and the combination of CHA_2_DS_2_-VASc score with LA volume index and LA positive strain or negative strain rate in the four-chamber view had the best predictive ability for LAAT (*χ*^2^: 33.47 and 33.48, respectively). We propose two novel and simple models for noninvasive LAAT prediction in patients with chronic nonvalvular atrial fibrillation. These models combine the CHA_2_DS_2_-VASc score with LA volume index and LA longitudinal strain parameters measured on speckle tracking echocardiography in the four-chamber view. We hope these simple models can help with decision-making in managing the antithrombotic treatment of such patients, whose risk of stroke cannot be determined solely based on the CHA_2_DS_2_-VASc score.

## 1. Introduction

Atrial fibrillation (AF) is the most prevalent arrhythmia in the general population, especially among the elderly [1]. In European countries, the prevalence of AF ranges from 1.9% in Italy, Iceland, and England, to 2.3% in Germany, and 2.9% in Sweden. These numbers are expected to increase in the near future [2]. The prevalence of AF is lower in Asian than in Western countries, having been reported at approximately 1.6% in Japan [3] and 1.0% in Korea [4]. AF is associated with increased risk of ischemic stroke, systemic thromboembolism, and transient ischemic attack, which ranges from 1.5% in individuals aged 50–59 years to 23.5% in individuals aged 80–89 years [5]. Recent studies indicate that cardioembolic stroke accounts for 16%–30% of cases of ischemic stroke [6]. Compared to non-AF stroke, cardioembolic stroke due to AF is associated with higher risk of mortality and worse outcomes [7]. Therefore, it is of utmost importance to clarify the risk factors for AF stroke and adequately stratify patients according to stroke risk.

The left atrium (LA) is a specialized cardiac structure that generates and maintains chaotic electrical impulses in AF, eventually losing contractility and causing atrial hemodynamic instability. The LA has a distinctive appendage shaped as a finger-like pouch extending from the main body of the LA and is considered the main source of clot formation in AF [8]. Therefore, it is important to adequately assess the LA appendage.

While cardiac computer tomography and magnetic resonance imaging are the most accurate methods for LA assessment, these methods are expensive and time-consuming and moreover require highly qualified expertise. Speckle tracking echocardiography (STE) is a new ultrasound-based modality designed for left ventricular assessment. However, recent studies have shown that STE can also be applied for measuring LA strain and strain rate [9–11]. Therefore, STE may be useful in screening AF patients for stroke risk.

Several STE-measurable parameters including LA diameter, LA volume index (LAVi), LA strain, and LA strain rate are known to be independently associated with stroke in AF patients [12–14]. However, the association of such parameters with thromboembolism in AF remains unclear. Therefore, in this study, we aimed to develop simple models for predicting the presence of LA appendage thrombus (LAAT) based on clinical factors and LA parameters measured on STE. Such models may help with decision-making in managing the antithrombotic treatment of patients with chronic nonvalvular AF.

## 2. Materials and Methods

### 2.1. Patients

From September 2013 to December 2017, we intentionally enrolled 144 anticoagulant-naïve patients aged 40–90 years and diagnosed with permanent nonvalvular AF defined according to the guidelines issued by the European Society of Cardiology in 2010 [15]. We excluded patients with moderate-to-severe rheumatic mitral stenosis with regurgitation, aortic stenosis with regurgitation, prosthetic valve, or previous surgical valvular repair. All patients underwent transesophageal echocardiography (TEE) to check for LAAT.

The study protocol was approved by our hospital's ethics committee, and all patients received extensive explanations regarding the risks associated with TEE. Informed consent was obtained from all patients before the procedure.

All patients were treated with AF rate control drugs to get their heart rates from 50 to 90 beats per minute and were carefully examined. The CHA_2_DS_2_-VASc score was calculated as follows: congestive heart failure, 1 point; hypertension, 1 point; age ≥75 years, 2 points; diabetes type 2, 1 point; stroke or history of transient ischemic attack, 2 points; vascular disease (i.e., prior myocardial infarction, peripheral artery disease, or aortic plaque), 1 point; age 65–74 years, 1 point; female sex, 1 point.

### 2.2. Transthoracic Echocardiography Evaluation

Echocardiography was performed using a high-quality ultrasound machine (VIVID 7; GE Medical Systems, Milwaukee, WI, USA) equipped with a 1.7/3.4-MHz tissue harmonic transducer. Electrocardiograms were recorded for all patients and synchronized with the echocardiographic images. Gain, depth, and focus position were adjusted to obtain a frame rate of 40–60 frames per second. Ultrasound beam width was set to obtain clear endocardial borders of the left ventricle and LA for offline processing and analysis. All images were acquired at the end of the expiratory period for three consecutive cardiac cycles and stored on a hard disk. All measurements were conducted with the patient in the left lateral decubitus position. STE data were analyzed using dedicated software. Speckle tracking and cardiac chamber measurements were conducted according to the guidelines issued by the American Society of Echocardiography and the European Association of Cardiovascular Imaging [16–19].

In the four- and two-chamber views, LA volume (mL) was measured by tracing the endocardium and applying the area-length technique. Specifically, LA volume was calculated as 8/(3*π*) [(A1·A2)/*L*], where A1 is the LA area (cm^2^) in the four-chamber view, A2 is the LA area (cm^2^) in the two-chamber view, and *L* (cm) is the shortest of the two long axes. Subsequently, the LAVi (mL/m^2^) was obtained as the LA volume (mL) divided by the body surface area (m^2^).

**Figure 1:**
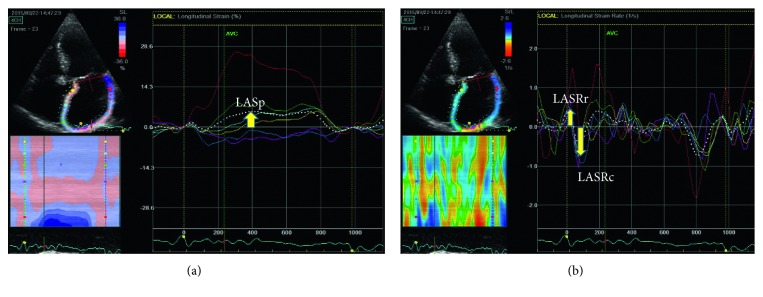
Measurement of left atrial (LA) strain and strain rate in the four-chamber view. (a) LA positive strain (LASp). (b) Evaluation of LA longitudinal strain rate was conducted separately for positive (LASRr) and negative (LASRc) values.

LA strain and strain rate were measured for the four- and two-chamber views of images obtained using two-dimensional STE and analyzed using EchoPAC (GE Medical Systems). EchoPAC was designed for echocardiographic assessment of the left ventricle [19]. However, recent studies demonstrated that EchoPAC can also be applied for LA speckle tracking analysis [20, 21]. After selecting an echo image at a specific cardiac cycle, the LA endocardium was traced manually from the mitral valve ring to the opposite end, ignoring the pulmonary vein. While epicardial borders could be detected automatically, some adjustments were sometimes necessary to cover the entire LA wall thickness. Before initiating image processing, the software requested the operator to confirm the selections and regions of interest. For each view, the software divided the LA into six segments (different color coding in Figure 1). The LA strain and strain rate were obtained separately for each segment and then averaged over all 12 segments.

### 2.3. TEE Evaluation

TEE was performed using the same ultrasound machine (VIVID 7; GE Medical Systems) equipped with the 3.5/7-MHz multiplane probe. Patients fasted for at least 6 hours received local anesthesia using lignocaine spray and, if necessary, intravenous midazolam (3–5 mg) for sedation. With the patients in the left lateral decubitus position, the transducer was slowly advanced through the mouth guard into the esophagus. If any resistance was met, the direction of the probe was carefully shifted and advancement was resumed. If it was not possible to advance the probe, the procedure was stopped.

All cardiac chambers were surveyed carefully to search for thrombus in the LA and LA appendage. Thrombus was defined as a fixed or mobile echogenic mass clearly distinguishable from the wall of the LA or LA appendage. Spontaneous echo contrast was diagnosed as dynamic or “smoke-like” echo signal inside the LA that could not be eliminated by changing the gain settings [22].

### 2.4. Reproducibility

Interobserver variablity and intraobserver variability for LA strain and strain rate were studied twice in a group of 10 randomly selected subjects by one operator and by two investigators who were unaware of the previous results. The coefficient of variation for positive strain in the four-chamber view was 10.2% (intraobserver) and 18.8% (interobserver) and for positive strain rate in four-chamber view was 9.4% (intraobserver) and 13.7% (interobserver).

### 2.5. Statistical Analysis

Continuous variables are expressed as mean ± standard deviation, while discrete variables are expressed as frequency (percentage). All echocardiographic variables were tested for normality using the Kolmogorov–Smirnov test. Between-group comparisons were conducted using the Student's *t*-test for data with normal distribution and using the Mann–Whitney test for data with nonnormal distribution. Logistic regression models were used to evaluate the association between binary and continuous variables and examine the performance of different predictive models. Receiver operating characteristic (ROC) curves were analyzed to identify the optimal cutoff values of echocardiographic variables for predicting the presence of LAAT, and the results were expressed as the area under the ROC curve (AUC). The quality of the models was expressed in terms of odds ratios (ORs) with 95% confidence intervals (95% CIs). Bland–Altman analysis was conducted to assess intraobserver and interobserver variability. *p* values ≤0.05 were considered to indicate statistical significance.

## 3. Results

### 3.1. Characteristics of the Study Population

From September 2013 to December 2017, we enrolled 181 patients who had no history of using antithrombotic or antiplatelet therapy. However, 37 (20.4%) patients were excluded from the study because of unacceptable quality of transthoracic images. All remaining patients (*n* = 144) underwent TEE successfully. Among these, 35 (24.3%) patients were found to have LAAT. The demographic, clinical, and left ventricular echocardiographic characteristics of the 144 patients included in the final analysis are summarized in Table 1.

Upon stratifying the patients according to the presence of LAAT (with vs without LAAT), the two groups did not differ in terms of average age, body mass index, or left ventricular dimensions, volume, and ejection fraction. However, the prevalence of hypertension was higher among patients with LAAT than among those without LAAT (82.9% vs 64.2%), as was the prevalence of ischemic stroke (45.7% vs 23.9%). Other risk factors (vascular diseases, heart failure, and diabetes) did not differ between the two groups.

### 3.2. LA Parameters

We found that LA parameters on echocardiography (diameter, area, volume, and volume index) were larger in nonvalvular AF patients with LAAT than in those without LAAT. On the contrary, LA parameters on STE (positive strain, positive strain rate, and negative strain rate in the two- and four-chamber views) were lower in patients with LAAT than in those without LAAT. All differences were statistically significant (Table 2).

We conducted ROC curve analysis to evaluate the diagnostic value of LA echocardiographic parameters for LAAT prediction in patients with chronic nonvalvular AF. The highest diagnostic performance was noted for LA positive strain in the four-chamber view (optimal cutoff, ≤6.7%; AUC = 0.84; sensitivity = 77.1%; specificity = 77.1%), whereas the lowest performance was noted for LA anterior-posterior diameter (optimal cutoff, >5.7 cm; AUC = 0.65; sensitivity = 94.3%; specificity = 32.1%). The results of the ROC curve analysis for all LA parameters are summarized in Table 3. All LA parameters had good AUC, ranging from 0.71 (LA area in the four-chamber view >26.7 cm^2^; LA positive strain rate in the two-chamber view ≤0.58 s^−1^; LA negative strain rate in the four-chamber view >−0.94 s^−1^) to 0.83 (LA negative strain rate in the four-chamber view >−0.73 s^−1^).

**Figure 2:**
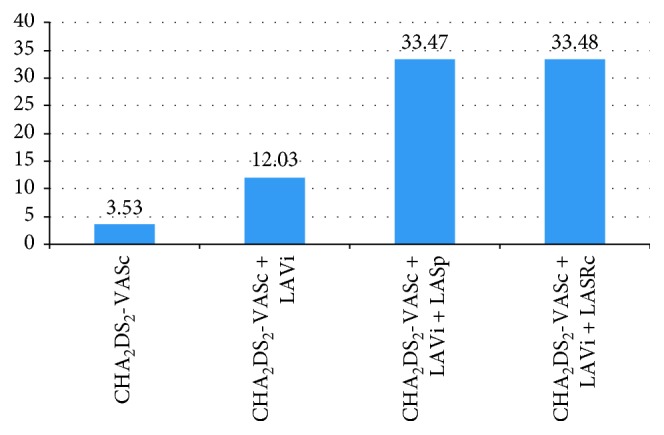
Performance of predictive models based on the CHA_2_DS_2_-VASc score and left atrial speckle tracking parameters for LAAT prediction.

Using logistic regression analysis and ROC curve analysis, we were able to test several models for LAAT prediction (Figure 2). The model based solely on the CHA_2_DS_2_-VASc score (model 1) revealed no association between this parameter and LAAT (OR = 1.24; 95% CI, 0.99–1.55), providing low predictive ability (*χ*^2^ = 3.53). The model considering the LAVi in addition to the CHA_2_DS_2_-VASc score (model 2) revealed a significant association between LAAT and the combination of these parameters (OR = 1.03; 95% CI, 1.01–1.05; *p* < 0.01), providing higher predictive ability (*χ*^2^ = 12.03). Adding STE-based LA parameters further increased the quality of the predictive models for LAAT detection. Specifically, models involving the CHA_2_DS_2_-VASc score, LAVi, and LA positive strain in the four-chamber view (model 3) or LA negative strain rate in the two-chamber view (model 4) revealed significant associations between LAAT and these combinations of parameters, providing high but comparable predictive ability (*χ*^2^: 33.47 and 33.48, respectively) (Table 4).

## 4. Discussion

Many patients referred to our tertiary hospital for chronic AF had no history of antithrombotic or antiplatelet therapy, which often means that the patients were not aware of their disorder or that their general practitioners did not prescribe adequate therapy based on the CHA_2_DS_2_-VASc score [23, 24]. Moreover, this group of patients often presented with persistent episodes of atrial tachyarrhythmia which were associated with elevated asymmetric dimethylarginine (ADMA) and downregulates endothelial nitric oxide synthase (eNOS). These factors were shown to be the risks of oxidative stress, vascular injury, and endothelial dysfunction [25]. This might be the reason why the prevalence of LAAT was higher in our study (24.3%) than in the previous studies [26, 27].

Thromboembolism is the most common complication in patients with chronic nonvalvular AF. While TEE is the gold standard tool to detect LAAT [28–31], this modality is semi-invasive and may not be applicable in all cases. Recent studies reported the successful use of noninvasive approaches to diagnose LAAT, including two-dimensional STE-based measurement of LA parameters [32, 33]. However, these models are complex and not easy to apply in clinical practice. Therefore, we set out to develop simple predictive models for LAAT detection.

Recent guidelines recommend using the CHA_2_DS_2_-VASc score to predict stroke risk in AF patients [15, 34], as high CHA_2_DS_2_-VASc score (≥4) was shown to be associated with thromboembolism in nonvalvular AF patients [35]. However, in our study, the mean CHA_2_DS_2_-VASc score in both groups was below 4 (with LAAT, 3.63 ± 2.0; without LAAT, 3.0 ± 1.6; Table 1). In other words, the CHA_2_DS_2_-VASc score alone cannot be used to predict LAAT in treatment-naïve patients with chronic nonvalvular AF (see also Figure 2). Therefore, we tried to increase the predictive value of CHA_2_DS_2_-VASc score-based models by adding LA echocardiographic parameters. Indeed, the combination of CHA_2_DS_2_-VASc score with LA parameters, especially with strain parameters, had a significant association with LAAT and provided high predictive ability (models 3 and 4; Figure 2).

Upon ROC curve analysis, we found good diagnostic performance for LA positive longitudinal strain in the four-chamber view (optimal cutoff, ≤6.7%; AUC = 0.84; sensitivity = 77.1%; specificity = 77.1%), negative longitudinal strain rate in the four-chamber view (optimal cutoff, ≥0.73 s^−1^; AUC = 0.83; sensitivity = 85.7%; specificity = 70.6%), and LAVi (optimal cutoff, ≥57 mL/m^2^; AUC = 0.72; sensitivity = 77.1%; specificity = 64.5%). These results suggest that the diagnostic performance for LAAT detection in patients with chronic nonvalvular AF may be higher when using the combination of such LA-based parameters than when using the CHA_2_DS_2_-VASc score alone.

We found that the CHA_2_DS_2_-VASc score was not associated with LAAT (Figure 2), which is in agreement with the findings of Sugiura et al. [36] and Tang et al. [37]. However, by adding LAVi to the CHA_2_DS_2_-VASc score, we could increase the predictive ability of the model (*χ*^2^) from 3.53 to 12.53. Further addition of an LA strain parameter provided a substantial increase in predictive power (to 33.47 with the addition of LA positive strain, and to 33.48 with the addition of LA negative strain rate). These results can be explained by the fact that, in chronic nonvalvular AF, LA dilatation and dysfunction is the primary contributor to clot formation [38]. Other authors also proposed models combining clinical and echocardiographic parameters. For example, Obokata et al. proposed models that combined CHA_2_DS_2_-VASc score, oral anticoagulant use, left ventricular ejection fraction, and LA total longitudinal strain to increase predictive value [33]. The advantage of the models proposed in our present study is that they achieve good predictive ability using only echocardiographic parameters, thus being easier to apply in clinical practice.

There are several limitations to our study. Firstly, this was a single-center study enrolling a small number of patients without anticoagulation treatment. Secondly, the software we used to measure LA dimensions and strain (EchoPAC; GE Medical Systems) was specifically designed for evaluating the left ventricle, not the LA. Lastly, STE parameters vary across ultrasound machine manufacturers, which may preclude direct comparison with previously reported values.

## 5. Conclusions

We propose two novel and simple models for noninvasive LAAT prediction in patients with chronic nonvalvular AF. These models combine the CHA_2_DS_2_-VASc score with LAVi and LA longitudinal strain parameters (positive strain or negative strain rate) measured on STE in the four-chamber view. We hope these simple models can help with decision-making in managing the antithrombotic treatment of such patients, whose risk of stroke cannot be determined solely based on the CHA_2_DS_2_-VASc score.

## Figures and Tables

**Table 1:** Demographic, clinical, and echocardiographic characteristics of patients with nonvalvular atrial fibrillation.

Characteristic	With LAAT (*n* = 35)	Without LAAT (*n* = 109)	*p* value
Female sex	6 (11.7%)	24 (22%)	<0.001
Age, years	72.4 ± 11.5	68.4 ± 10.6	0.20
BMI (kg/m^2^)	22.1 ± 2.1	22.5 ± 3.1	0.01
Heart rate (bpm)	76.3 ± 10.5	78.4 ± 11.5	0.33
Heart failure	10 (28.6%)	31 (28.4%)	<0.001
Hypertension	29 (82.9%)	70 (64.2%)	<0.001
Diabetes type 2	4 (11.4%)	17 (15.6%)	<0.001
History of ischemic stroke	16 (45.7%)	26 (23.9%)	<0.001
Vascular disease	7 (20%)	20 (18.3%)	<0.001
CHA_2_DS_2_-VASc score	3.63 ± 2.0	3.0 ± 1.6	0.08
LV diastolic diameter (mm)	50.4 ± 7.2	49.7 ± 8.5	0.31
LV systolic diameter (mm)	36.8 ± 8.2	35.3 ± 9.3	0.29
LV diastolic volume (mL)	124.7 ± 43	121.3 ± 51.6	0.47
LV systolic volume (mL)	62 ± 34.8	58.2 ± 4.04	0.36
LV ejection fraction (%)	47.1 ± 11.6	56 ± 13.6	0.16

Data represent frequency (percentage) or mean ± standard deviation. BMI: body mass index; LAAT: left atrial appendage thrombus; LV: left ventricle.

**Table 2:** Left atrial strain and strain rate parameters in patients with nonvalvular atrial fibrillation.

LA parameter on TTE	With LAAT (*n* = 35)	Without LAAT (*n* = 109)	*p* value
Diameter, cm	6.49 ± 0.59	6.13 ± 0.77	0.015
Area in two-chamber view (cm^2^)	28.51 ± 4.17	24.33 ± 5.54	<0.001
Area in four-chamber view (cm^2^)	30.0 ± 5.32	26.35 ± 5.43	0.001
Volume (mL)	111.79 ± 30.42	88.33 ± 29.02	<0.001
Volume index (mL/m^2^)	69.59 ± 21.17	55.78 ± 20.16	0.001
Positive strain in two-chamber view (%)	5.68 ± 1.91	10.61 ± 6.92	<0.001
Positive strain in four-chamber view (%)	5.28 ± 2.54	10.47 ± 5.31	<0.001
Positive strain rate in two-chamber view (s^−1^)	0.52 ± 0.16	0.7 ± 0.31	<0.001
Positive strain rate in four-chamber view (s^−1^)	0.51 ± 0.15	0.69 ± 0.26	<0.001
Negative strain rate in two-chamber view (s^−1^)	−0.63 ± 0.19	−0.91 ± 0.45	<0.001
Negative strain rate in four-chamber view (s^−1^)	−0.57 ± 0.18	−0.98 ± 0.41	<0.001

Data represent mean ± standard deviation. LA: left atrial; LAAT: left atrial appendage thrombus; TEE: transthoracic echocardiography.

**Table 3:** Diagnostic value of LA echocardiographic parameters for LAAT prediction.

LA parameter	Cutoff value	Sensitivity (%)	Specificity (%)	AUC	95% CI	*p* value
Area in four-chamber view (cm^2^)	>26.7	74.3	63.3	0.71	0.63–0.78	<0.001
Area in two-chamber view (cm^2^)	>26.8	71.4	75.2	0.76	0.68–0.83	<0.001
Diameter (cm)	>5.7	94.3	32.1	0.65	0.57–0.73	<0.001
Volume (mL)	>99.8	68.6	73.4	0.73	0.65–0.80	<0.001
Volume index (mL/m^2^)	>57.0	77.1	64.2	0.72	0.64–0.79	<0.001
Positive strain in two-chamber view (%)	≤7.81	91.4	67.0	0.81	0.74–0.87	<0.001
Positive strain in four-chamber view (%)	≤6.7	77.1	77.1	0.84	0.76–0.89	<0.001
Positive strain rate in two-chamber view (s^−1^)	≤0.58	74.3	60.6	0.71	0.63–0.78	<0.001
Positive strain rate in four-chamber view (s^−1^)	≤0.55	65.7	72.5	0.75	0.67–0.82	<0.001
Negative strain rate in two-chamber view (s^−1^)	>−0.94	100	44.0	0.71	0.63–0.78	<0.001
Negative strain rate in four-chamber view (s^−1^)	>−0.73	85.7	70.6	0.83	0.76–0.89	<0.001

Data were obtained using receiver operating characteristic curve analysis. 95% CI: 95% confidence interval; AUC: area under the curve; LA: left atrial; LAAT: left atrial appendage thrombus.

**Table 4:** Models of combined CHA2DS2-VASc score and left atrial speckle tracking parameters for LAA thrombus prediction.

*χ* ^2^	Model 1 (*χ*^2^ = 3.53)	Model 2 (*χ*^2^ = 12.03)	Model 3 (*χ*^2^ = 33.47)	Model 4 (*χ*^2^ = 33.48)
Variables	CHA_2_DS_2_-VASc	CHA_2_DS_2_-VASc + LAVi	CHA_2_DS_2_-VASc + LAVi + LASp(4C)	CHA_2_DS_2_-VASc + LAVi + LASRc(2C)
	OR = 1.24 (95% CI: 0.99–1.55)	OR = 1.15 (95% CI: 0.91–1.46)	OR = 1.10 (95% CI: 0.85–1.43)	OR = 1.10 (95% CI: 0.85–1.43)
	—	**OR** **=** **1.03 (95% CI: 1.01–1.05;p<0.01)**	OR = 1.02 (95% CI: 0.85–1.43)	OR = 1.02 (95% CI: 0.85–1.43)
	—	—	**OR** **=** **0.76 (95% CI: 0.66–0.68;p<0.01)**	**OR** **=** **0.76 (95% CI: 0.65-0.90;p<0.01)**
	—	—	—	**OR** **=** **0.82 (95% CI: 0.04–6.01;p<0.01)**

95% CI: 95% confidence interval; LAVi: left atrial volume index; LASp(4C): left atrial positive strain in four-chamber view; LASRc(2C): left atrial negative strain rate in two-chamber view; OR: odds ratio.

## Data Availability

The data used to support the findings of this study are available from the corresponding author upon request.

## References

[1] Zoni-Berisso M., Lercari F., Carazza T., Domenicucci S. (2014). Epidemiology of atrial fibrillation: European perspective. *Clinical Epidemiology*.

[2] Go A. S., Hylek E. M., Phillips K. A. (2001). Prevalence of diagnosed atrial fibrillation in adults. *JAMA*.

[3] Iguchi Y., Kimura K., Aoki J. (2008). Prevalence of atrial fibrillation in community-dwelling Japanese aged 40 Years or older in Japan. *Circulation Journal*.

[4] Lee K. S., Choi S. J., Park S. H. (2008). Prevalence of atrial fibrillation in middle-aged people in Korea: the Korean genome and epidemiology study. *Korean Circulation Journal*.

[5] Wolf P. A., Abbott R. D., Kannel W. B. (1991). Atrial fibrillation as an independent risk factor for stroke: the Framingham study. *Stroke*.

[6] Markus H. S., Khan U., Birns J. (2007). Differences in stroke subtypes between black and white patients with stroke. *Circulation*.

[7] Kirchhof P., Auricchio A., Bax J. (2007). Outcome parameters for trials in atrial fibrillation: executive summary: recommendations from a consensus conference organized by the German Atrial Fibrillation Competence NETwork (AFNET) and the European Heart Rhythm Association (EHRA). *European Heart Journal*.

[8] Ho S. Y., McCarthy K. P., Faletra F. F. (2011). Anatomy of the left atrium for interventional echocardiography. *European Journal of Echocardiography*.

[9] Faustino A., Providencia R., Barra S. (2014). Which method of left atrium size quantification is the most accurate to recognize thromboembolic risk in patients with non-valvular atrial fibrillation?. *Cardiovasc Ultrasound*.

[10] Vianna-Pinton R., Moreno C. A., Baxter C. M., Lee K. S., Tsang T. S., Appleton C. P. (2009). Two-dimensional speckle-tracking echocardiography of the left atrium: feasibility and regional contraction and relaxation differences in normal subjects. *Journal of the American Society of Echocardiography*.

[11] To A. C. Y., Flamm S. D., Marwick T. H., Klein A. L. (2011). Clinical utility of multimodality LA imaging. *JACC: Cardiovascular Imaging*.

[12] Abhayaratna W. P., Seward J. B., Appleton C. P. (2006). Left atrial size. *Journal of the American College of Cardiology*.

[13] Dittrich H. C., Pearce L. A., Asinger R. W. (1999). Left atrial diameter in nonvalvular atrial fibrillation: an echocardiographic study. *American Heart Journal*.

[14] Islas F., Olmos C., Vieira C. (2015). Thromboembolic risk in atrial fibrillation: association between left atrium mechanics and risk scores. A study based on 3D wall-motion tracking technology. *Echocardiography*.

[15] Camm A. J., Kirchhof P., Lip G. Y. (2010). Guidelines for the management of atrial fibrillation: the task force for the management of atrial fibrillation of the european society of Cardiology (ESC). *EP Europace*.

[16] Donal E., Lip G. Y. H., Galderisi M. (2016). EACVI/EHRA Expert Consensus Document on the role of multi-modality imaging for the evaluation of patients with atrial fibrillation. *European Heart Journal—Cardiovascular Imaging*.

[17] Lang R. M., Badano L. P., Mor-Avi V. (2015). Recommendations for cardiac chamber quantification by echocardiography in adults: an update from the American society of echocardiography and the European association of cardiovascular imaging. *Journal of the American Society of Echocardiography*.

[18] Mor-Avi V., Lang R. M., Badano L. P. (2011). Current and evolving echocardiographic techniques for the quantitative evaluation of cardiac mechanics: ASE/EAE consensus statement on methodology and indications endorsed by the Japanese society of echocardiography. *European Journal of Echocardiography*.

[19] Voigt J.-U., Pedrizzetti G., Lysyansky P. (2015). Definitions for a common standard for 2D speckle tracking echocardiography: consensus document of the EACVI/ASE/Industry task force to standardize deformation imaging. *Journal of the American Society of Echocardiography*.

[20] Motoki H., Dahiya A., Bhargava M. (2012). Assessment of left atrial mechanics in patients with atrial fibrillation: comparison between two-dimensional speckle-based strain and velocity vector imaging. *Journal of the American Society of Echocardiography*.

[21] Saraiva R. M., Demirkol S., Buakhamsri A. (2010). Left atrial strain measured by two-dimensional speckle tracking represents a new tool to evaluate left atrial function. *Journal of the American Society of Echocardiography*.

[22] Fatkin D., Kelly R. P., Feneley M. P. (1994). Relations between left atrial appendage blood flow velocity, spontaneous echocardiographic contrast and thromboembolic risk in vivo. *Journal of the American College of Cardiology*.

[23] Fuster V., Ryden L. E., Cannom D. S. (2011). ACCF/AHA/HRS focused updates incorporated into the ACC/AHA/ESC 2006 guidelines for the management of patients with atrial fibrilation: a report of the American College of Cardiology Foundation/American Heart Association Task Force on practice guidelines. *Circulation*.

[24] Kirchhof P., Benussi S., Kotecha D. (2016). ESC Guidelines for the management of atrial fibrillation developed in collaboration with EACTS. *EP Europace*.

[25] Goette A., Hammwöhner M., Bukowska A. (2012). The impact of rapid atrial pacing on ADMA and endothelial NOS. *International Journal of Cardiology*.

[26] Malik R., Alyeshmerni D. M., Wang Z. (2015). Prevalence and predictors of left atrial thrombus in patients with atrial fibrillation: is transesophageal echocardiography necessary before cardioversion?. *Cardiovascular Revascularization Medicine*.

[27] Corrado G., Beretta S., Sormani L. (2004). Prevalence of atrial thrombi in patients with atrial fibrillation/flutter and subtherapeutic anticoagulation prior to cardioversion. *European Journal of Echocardiography*.

[28] Hahn R. T., Abraham T., Adams M. S. (2013). Guidelines for performing a comprehensive transesophageal echocardiographic examination: recommendations from the American society of echocardiography and the society of cardiovascular anesthesiologists. *Journal of the American Society of Echocardiography*.

[29] Agmon Y., Khandheria B. K., Gentile F., Seward J. B. (1999). Echocardiographic assessment of the left atrial appendage. *Journal of the American College of Cardiology*.

[30] Leung D. Y., Davidson P. M., Cranney G. B., Walsh W. F. (1997). Thromboembolic risks of left atrial thrombus detected by transesophageal echocardiogram. *The American Journal of Cardiology*.

[31] Manning W. J., Weintraub R. M., Waksmonski C. A. (1995). Accuracy of transesophageal echocardiography for identifying left atrial thrombi: a prospective, intraoperative study. *Annals of Internal Medicine*.

[32] Leong D. P., Joyce E., Debonnaire P. (2017). Left atrial dysfunction in the pathogenesis of cryptogenic stroke: novel insights from speckle-tracking echocardiography. *Journal of the American Society of Echocardiography*.

[33] Obokata M., Negishi K., Kurosawa K. (2014). Left atrial strain provides incremental value for embolism risk stratification over CHA2DS2-VASc score and indicates prognostic impact in patients with atrial fibrillation. *Journal of the American Society of Echocardiography*.

[34] January C. T., Wann L. S., Alpert J. S. (2014). 2014 AHA/ACC/HRS guideline for the management of patients with atrial fibrillation: executive summary. *Circulation*.

[35] Zhang E., Liu T., Li Z., Zhao J., Li G. (2015). High CHA2DS2−VASc score predicts left atrial thrombus or spontaneous echo contrast detected by transesophageal echocardiography. *International Journal of Cardiology*.

[36] Sugiura S., Fujii E., Senga M., Sugiura E., Nakamura M., Ito M. (2012). Clinical features of patients with left atrial thrombus undergoing anticoagulant therapy. *Journal of Interventional Cardiac Electrophysiology*.

[37] Tang R.-B., Dong J.-Z., Yan X.-L. (2014). Serum uric acid and risk of left atrial thrombus in patients with nonvalvular atrial fibrillation. *Canadian Journal of Cardiology*.

[38] Lip G. Y. H., Rumley A., Dunn F. G., Lowe G. D. O. (1996). Thrombogenesis in mitral regurgitation and aortic stenosis. *Angiology*.

